# Inhibiting cardiac myeloperoxidase alleviates the relaxation defect in hypertrophic cardiomyocytes

**DOI:** 10.1093/cvr/cvab077

**Published:** 2021-03-11

**Authors:** Chrishan J A Ramachandra, Myu Mai Ja Kp, Jasper Chua, Sauri Hernandez-Resendiz, Elisa A Liehn, Ralph Knöll, Li-Ming Gan, Erik Michaëlsson, Malin K B Jonsson, Katarina Ryden-Markinhuhta, Ratan V Bhat, Regina Fritsche-Danielson, Ying-Hsi Lin, Sakthivel Sadayappan, Hak Chiaw Tang, Philip Wong, Winston Shim, Derek J Hausenloy

**Affiliations:** 1 National Heart Research Institute Singapore, National Heart Centre Singapore, Singapore, Singapore; 2 Cardiovascular & Metabolic Disorders Program, Duke-National University of Singapore Medical School, Singapore, Singapore; 3 Faculty of Science, National University of Singapore, Singapore, Singapore; 4 Bioscience, Cardiovascular, Renal & Metabolism, BioPharmaceuticals R&D, AstraZeneca, Gothenburg, Sweden; 5 Department of Medicine (MedH), Integrated Cardio Metabolic Centre (ICMC), Heart and Vascular Theme, Karolinska Institute, Stockholm SE-171 77, Sweden; 6 Early Clinical Development, Research and Early Development Cardiovascular, Renal and Metabolism (CVRM), BioPharmaceuticals R&D, AstraZeneca, Gothenburg, Sweden; 7 Bioscience Cardiovascular, Research and Early Development Cardiovascular, Renal and Metabolism (CVRM), BioPharmaceuticals R&D, AstraZeneca, Gothenburg, Sweden; 8 Research and Early Development Cardiovascular, Renal and Metabolism (CVRM), BioPharmaceuticals R&D, AstraZeneca, Gothenburg, Sweden; 9 Division of Cardiovascular Health and Disease, Department of Internal Medicine, Heart, Lung and Vascular Institute, University of Cincinnati, Cincinnati, OH, USA; 10 Department of Cardiology, National Heart Centre Singapore, Singapore, Singapore; 11 Health and Social Sciences Cluster, Singapore Institute of Technology, Singapore, Singapore; 12 Yong Loo Lin School of Medicine, National University of Singapore, Singapore, Singapore; 13 The Hatter Cardiovascular Institute, University College London, London, UK; 14 Cardiovascular Research Center, College of Medical and Health Sciences, Asia University, Taichung, Taiwan

**Keywords:** Myeloperoxidase, Hypertrophic cardiomyopathy (HCM), Diastolic dysfunction, Human-induced pluripotent stem cells (hiPSCs), Cardiac myosin binding protein-C (MYBPC3), Oxidative stress

## Abstract

**Aims:**

Hypertrophic cardiomyopathy (HCM) is characterized by cardiomyocyte hypertrophy and disarray, and myocardial stiffness due to interstitial fibrosis, which result in impaired left ventricular filling and diastolic dysfunction. The latter manifests as exercise intolerance, angina, and dyspnoea. There is currently no specific treatment for improving diastolic function in HCM. Here, we investigated whether myeloperoxidase (MPO) is expressed in cardiomyocytes and provides a novel therapeutic target for alleviating diastolic dysfunction in HCM.

**Methods and results:**

Human cardiomyocytes derived from control-induced pluripotent stem cells (iPSC-CMs) were shown to express MPO, with MPO levels being increased in iPSC-CMs generated from two HCM patients harbouring sarcomeric mutations in the *MYBPC3* and *MYH7* genes. The presence of cardiomyocyte MPO was associated with higher chlorination and peroxidation activity, increased levels of 3-chlorotyrosine-modified cardiac myosin binding protein-C (MYBPC3), attenuated phosphorylation of MYBPC3 at Ser-282, perturbed calcium signalling, and impaired cardiomyocyte relaxation. Interestingly, treatment with the MPO inhibitor, AZD5904, reduced 3-chlorotyrosine-modified MYBPC3 levels, restored MYBPC3 phosphorylation, and alleviated the calcium signalling and relaxation defects. Finally, we found that MPO protein was expressed in healthy adult murine and human cardiomyocytes, and MPO levels were increased in diseased hearts with left ventricular hypertrophy.

**Conclusion:**

This study demonstrates that MPO inhibition alleviates the relaxation defect in hypertrophic iPSC-CMs through MYBPC3 phosphorylation. These findings highlight cardiomyocyte MPO as a novel therapeutic target for improving myocardial relaxation associated with HCM, a treatment strategy which can be readily investigated in the clinical setting, given that MPO inhibitors are already available for clinical testing.

## Graphical Abstract

**Figure:**
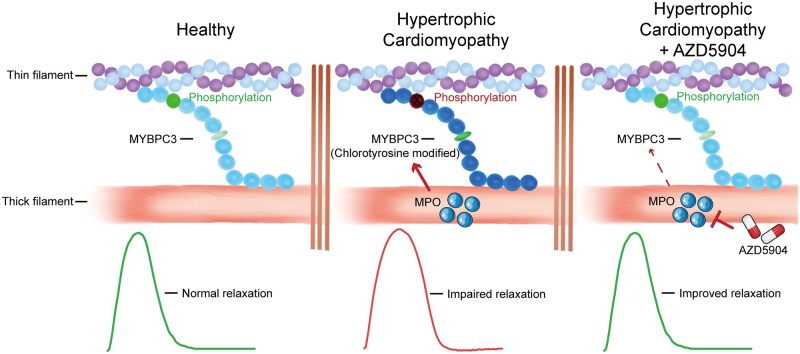

**Time for primary review: 18 days**

## 1. Introduction

Hypertrophic cardiomyopathy (HCM) is one of the most common inherited cardiac disease, affecting ∼1 in 500 individuals.^1^ It is defined as left ventricular hypertrophy (LVH) without chamber dilation, which develops in the absence of an identifiable cause. It is characterized by cardiomyocyte hypertrophy and disorganization, myofibre disarray, and interstitial fibrosis, which result in increased left ventricular (LV) stiffness, impaired LV filling and diastolic dysfunction, and manifests as dyspnoea and exercise intolerance.^2^^,^^3^ HCM is also the leading cause of sudden cardiac death in adolescents, young adults, and athletes, and this is mediated primarily by ventricular tachycardia and fibrillation due to the underlying arrhythmogenic substrate.^4^ HCM patients with limiting symptoms of exercise intolerance, angina, and dyspnoea, are often managed with β-blockers and L-type calcium channel blockers.^5^ However, these treatments do not directly address the underlying contractile impediment of diastolic dysfunction. Hence, there is an unmet need to identify novel therapeutic targets that can alleviate diastolic dysfunction and improve symptoms in patients with HCM.

Myeloperoxidase (MPO) is a member of the superfamily of haem peroxidases that is mainly expressed in neutrophils and monocytes, where it is stored in azurophilic granules. MPO is secreted upon leucocyte activation, and through enzymatic production of hypohalous acids and other reactive species, it post-translationally modifies different target proteins,^6^ and plays an important role in antimicrobial activity and innate immunity.^7^ Dysregulated MPO release can lead to tissue damage in various diseases.^8^^,^^9^ Increased circulating levels of MPO are indicative of underlying inflammation and oxidative stress, and have been associated with a wide variety of cardiovascular diseases (CVDs) including coronary artery disease, congestive heart failure, arterial hypertension, peripheral arterial disease, and cardiac arrhythmias. Elevated circulating MPO levels have been associated with poor prognosis and increased risk of CVD-related mortality.^10^ Although MPO is predominantly found in neutrophils and monocytes, it has also been shown to be expressed in other cell types including neurons^11^ and endothelial cells.^12^

In this study, we hypothesized that MPO is expressed in cardiomyocytes, and is up-regulated under conditions of cellular stress such as HCM, where it modifies sarcomere proteins and contributes to diastolic dysfunction. We evaluated cardiomyocytes derived from induced pluripotent stem cells (iPSC-CMs) from patients with mutations in *MYH7* and *MYBPC3*, the two most common gene mutations associated with HCM.^1^ We show for the first time the presence of functional MPO within iPSC-CMs, and have demonstrated that in iPSC-CMs from HCM patients (HCM-CMs), it reduced the phosphorylation of 3-chlorotyrosine-modified cardiac myosin binding protein-C (MYBPC3) protein and delayed cardiomyocyte relaxation. Importantly, treatment of HCM-CMs with the MPO inhibitor AZD5904^13^ restored phosphorylation of MYBPC3, and alleviated the cardiomyocyte relaxation defect, highlighting MPO inhibition as a novel therapy for improving diastolic function in HCM patients. Importantly, we also demonstrated MPO protein expression in cardiomyocytes from healthy and diseased hypertrophied adult murine and human hearts.

## 2. Methods

### 2.1 Ethics statement

The iPSC part of the study was approved by the SingHealth Centralized Institutional Review Board, reference number 2015/2521. Study participants gave informed consent prior to their inclusion in the study and all investigations conformed with the principles outlined in the Declaration of Helsinki. The human heart tissue part of the study was approved by the Ethics Committee from Medical Faculty, Rheinisch–Westfälische Technische Hochschule Aachen, Germany (reference number BUS EK 062/13). Human cardiac tissue specimens were obtained from post-mortem samples of patients with heart failure and advanced stages of LVH. Informed written consent for use of post-mortem tissue was obtained from the patient’s next-of-kin. All animal procedures were approved by the Institutional Animal Care and Use Committee, reference number 2020/SHS/156 and conformed to the guidelines from Directive 2010/63/EU of the European Parliament on the protection of animals used for scientific purposes.

### 2.2 Generation of human-induced pluripotent stem cells and cardiomyocyte differentiation

Control-induced pluripotent stem cells (iPSCs) were derived from healthy subjects reported previously.^14^ The HCM iPSC line harbouring a D389V missense mutation + Δ25 bp intronic deletion in the *MYBPC3* gene was a kind gift from Professor Sakthivel Sadayappan, University of Cincinnati, USA. The HCM iPSC line harbouring a R243C missense mutation in the *MYH7* gene was generated in-house from peripheral blood mononuclear cells, in accordance with the protocol described by Quintana-Bustamante and Segovia.^15^ All iPSC lines were maintained in mTeSR (Stemcell Technologies, Vancouver, Canada) under feeder-free conditions, with routine passaging every 7 days as reported previously.^16^ Human iPSCs were differentiated into cardiomyocytes using a previously described in-house protocol,^17^ which generates mature beating cardiac clusters by Day 30 post-differentiation.^18^

### 2.3 Transverse aortic constriction

Adult male C57Bl/6N mice (8–10 weeks old) were used in the study. Transverse aortic constriction (TAC) was performed by banding between the innominate and left carotid arteries. In brief, mice were intubated under general anaesthesia (intraperitoneal injection of 65 mg/kg ketamine, 13 mg/kg xylazine) and analgesia (intraperitoneal injection of 0.5 mg/kg Buprenorphine). The animals received mechanical ventilation (VentElite, Harvard apparatus) and ECG was carried out under anaesthesia to monitor heart rate. In sham control mice, the exact procedure was performed except for the ligation of the aorta. Animals were allowed to recover on a heated pad. Mice were monitored postoperatively for behavioural changes indicative of pain or distress. The successful ligation of the transverse aorta was confirmed by echocardiographic imaging using Vevo 2100 30-MHz microimaging system (Visual Sonics Inc., Toronto, Canada). At 1, 4, and 8 weeks, mice were anaesthetized using intraperitoneal injection of 65 mg/kg ketamine and 13 mg/kg xylazine. Upon the onset of deep anaesthesia, identified as the loss of the pedal pain withdrawal reflex, slowing of heart rate and breathing, mice were euthanized by excising the heart. The explanted hearts were washed in PBS, fixed with 10% neutral buffered formalin and embedded in paraffin after which tissues were sectioned at 4-µm thickness using Leica cryostat (Leica Biosystems, Wetzlar, Germany).

### 2.4 Immunofluorescence and immunohistochemistry

Cardiac clusters were dissociated into single cells using Embryoid Body Dissociation Kit (Miltenyi Biotec, Bergisch Gladbach, Germany), and seeded on 0.1% gelatin-coated glass bottom plates. Cells were fixed with 4% paraformaldehyde, permeabilized with 0.1% Triton X-100, blocked with 5% BSA and stained with primary antibodies overnight. Cells were washed, probed with respective fluorophore-conjugated secondary antibodies and counterstained with 4′,6-diamidino-2-phenylindole (DAPI) the following day. Mouse cardiac tissue staining was performed as follows. Slides containing paraffin embedded tissue were first placed in an oven (55–60°C) to melt the wax after which deparaffinization was performed using Histo-Clear. Rehydration of the tissue was performed following serial washes in ethanol (100%, 95%, 90%, 80%, and 70%) by transferring slides to water. Tissue was permeabilized with 0.025% Triton X-100, blocked with 10% horse serum and stained with primary antibody overnight. Tissue was washed and probed with respective fluorophore-conjugated secondary antibodies the following day and to quench autofluorescence, tissue was mounted in TrueVIEW (with DAPI; Vector Laboratories, CA, USA). Stained iPSC-CMs and mouse cardiac tissue (LV free wall) were examined under Zeiss LSM710 NLO multi-photon confocal microscope (Carl Zeiss Microscopy GmbH, Jena, Germany). Human cardiac tissue specimens were processed similarly to mouse tissue with the exception of Bloxall (Vector Laboratories) treatment following primary antibody incubation. Tissue was washed and probed with alkaline phosphatase-conjugated secondary antibody and subsequently treated with red substrate (Vector Laboratories) until colour developed. Finally, tissue was stained with haematoxylin (Vector Laboratories) and mounted and examined under Zeiss Axiovert 200 M microscope (Carl Zeiss Microscopy GmbH). Antibodies used in the study are listed in Supplementary material online, *Table S1*. For cell size and multi-nucleation analysis, cells were stained against α-actinin and counterstained with DAPI. Cell size was assessed using the wand tracing tool in ImageJ and multi-nucleation was assessed by visual counting.

### 2.5 Western blot, proteome profiler array and immunoprecipitation

Western blots were performed as described previously.^16^ Briefly, extracted proteins (25 µg) from cardiac clusters were subjected to electrophoresis and transferred to nitrocellulose (NC) membranes using iBlot Dry Blotting System (Thermo Fisher Scientific). NC membranes were blocked and probed with primary antibodies overnight. The following day, NC membranes were washed, probed with respective HRP-conjugated secondary antibodies and developed using SignalFire™ ECL Reagent (Cell Signalling Technology, MA, USA). The proteome profiler array (Human Pluripotent Stem Cell Array kit) was performed as per the manufacturer’s instructions using 150 µg of iPSC protein lysate (Research and Diagnostic Systems, Inc., MN, USA). Immunoprecipitation was performed as per the manufacturer’s instructions (Cell Signalling Technology). Briefly, 200 µg of cell lysate was incubated with the target antibody overnight, followed by incubation with agarose beads the next day. Agarose beads were washed and denatured samples were loaded on 4–12% Bis‐Tris Bolt Gels and western blot was performed as described above. Images were captured using C-DiGit Blot Scanner (LI-COR, NE, USA) and densitometric analysis was performed using Image Studio (LI-COR). Antibodies used in the study are listed in Supplementary material online, *Table S1*.

### 2.6 Real-time PCR and sequencing

For quantitative real-time PCR (RT-PCR), isolated RNA from cardiac clusters was converted to complementary DNA (cDNA) using SuperScript III First-Strand Synthesis System (Thermo Fisher Scientific, MA, USA) as previously reported.^19^ Using QuantiFast SYBR Green PCR Kit (Qiagen, Hilden, Germany) and RotorGene Q (Qiagen), cDNA templates were cycled as follows: 5 min at 95°C, followed by 40 cycles of 10 s at 95°C and 30 s at 60°C. Relative quantification was calculated according to ^ΔΔ^Ct method using GAPDH as endogenous control. For gene sequencing, using Platinum PCR SuperMix (Thermo Fisher Scientific) cDNA templates were cycled as follows: 2 min at 94°C, followed by 35 cycles of 30 s at 94°C, 30 s at 60°C, and 45 s at 72°C. Primers used in the study are listed in Supplementary material online, *Table S2*. Sanger sequencing services were provided by AITbiotech, Singapore.

### 2.7 Calcium transient and contraction analysis

For calcium transient analysis, cardiac clusters were dissociated into single cells and stained with Fluo-4, AM (Thermo Fisher Scientific) for 15 min at 37°C. Using C-Pace EP (IonOptix, MA, USA), cells were paced at either 0.25 or 0.5 Hz and calcium transients were recorded using MetaMorph Imaging System (Molecular Devices, CA, USA). Calcium transients were analysed using pCLAMP 10 (Molecular Devices). For contraction analysis, cardiac clusters were paced at 0.25 Hz using C-Pace EP (IonOptix) and video recordings were acquired using CKX41 Inverted Microscope (Olympus). The acquired videos were first analysed using Musclemotion^20^ and the raw data generated was then re-analysed using pCLAMP 10 (Molecular Devices).

### 2.8 MPO assessment and evaluation of oxidative stress

MPO was extracted and assayed from cardiac clusters using EnzChek MPO Activity Assay Kit as per the manufacturer’s instructions (Thermo Fisher Scientific). Chlorination and peroxidation activities were normalized to total protein content. Reactive oxygen species (ROS) levels were evaluated using H2DCFDA (Thermo Fisher Scientific). Cardiac clusters were dissociated into single cells using Embryoid Body Dissociation Kit (Miltenyi Biotec), and seeded on 0.1% gelatin-coated black clear-bottom 96-well plates. Cells were incubated with H2DCFDA in Hank's Balanced Salt Solution (HBSS) at 10 µM for 15 min at 37°C after which the probe was removed and cells were incubated for 30 min at 37°C in cardiomyocyte maintenance media.^18^ Maintenance media was then replaced with HBSS and fluorescence intensity was measured using Infinite 200 Microplate Reader (Tecan, Männedorf, Switzerland) at excitation 492 nm and emission 525 nm wavelengths. ROS levels were normalized to total protein content.

### 2.9 Statistical analysis

Data with normal Gaussian distribution were analysed by standard parametric tests and those with non-Gaussian distribution were analysed by non-parametric tests. The test used for each experiment is listed in the figure legends. A *P*-value of <0.05 was considered statistically significant.

## 3. Results

### 3.1 iPSC-CMs derived from HCM patients exhibit hypertrophic phenotype

A total of three iPSC lines were used in this study. One line was derived from a healthy individual which was reported previously^14^ and is hereafter referred to as control. Two other iPSC lines harbouring mutations in the *MYBPC3* and *MYH7* gene were also used in this study and are hereafter, referred to as HCM-1 and HCM-2, respectively. While HCM-1 harbours a heterozygous D389V missense mutation + Δ25 bp intronic deletion,^21^ HCM-2 has a heterozygous R243C missense mutation.^22^

**Figure 1:**
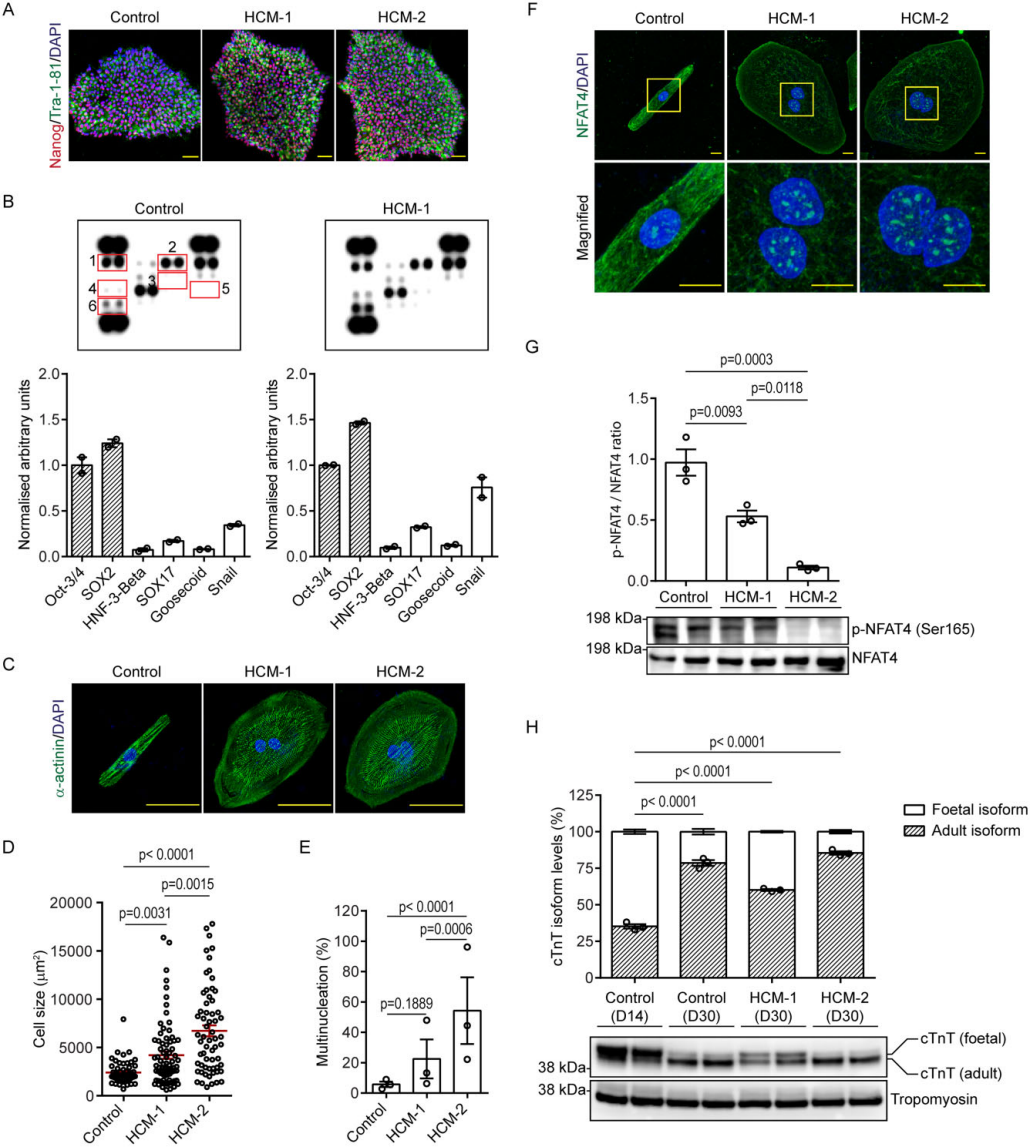
Morphological characterization of HCM-CMs. (*A*) Representative immunofluorescence pictographs of control- and HCM-iPSCs stained against pluripotency antigens; Nanog (red) and Tra-1-81 (green), and counterstained with DAPI (blue). Scale bar: 50 µm. (*B*) Proteome profiler array showing expression of pluripotent (shaded bars) and non-pluripotent markers (clear bars) in control and HCM-1 iPSCs with graphs presented as mean ± S.E.M. showing densitometry data normalized to internal control. Numbers: Oct-3/4 (1), SOX2 (2), HNF-3-Beta (3), SOX17 (4), Goosecoid (5), and Snail (6). (*C*) Representative immunofluorescence pictographs of control- and HCM-CMs stained against sarcomeric α-actinin (green) and counterstained with DAPI (blue). Scale bar: 50 µm. (*D*) Graph presented as mean ± S.E.M. showing comparisons in cell size between control- and HCM-CMs (Control *n* = 54; HCM-1 *n* = 76; HCM-2 *n* = 62; Kruskal–Wallis followed by Dunn’s *post hoc* test; three independent experiments). (*E*) Graph presented as mean ± S.E.M. showing percentage of multinucleated cells in control- and HCM-CMs (Control *n* = 70; HCM-1 *n* = 93; HCM-2 *n* = 83; one-way analysis of variance (ANOVA) followed by Tukey’s *post hoc* test; three independent experiments). (*F*) Representative immunofluorescence pictographs of control- and HCM-CMs stained against NFAT4 (green) and counterstained with DAPI (blue). Yellow inset represents magnified region. Scale bar: 10 µm. (*G*) Western blot showing phosphorylated NFAT4 levels in control- and HCM-CMs, with graphs presented as mean ± S.E.M. showing densitometry data normalized to total NFAT4 (*n* = 3 independent experiments; one-way ANOVA followed by Tukey’s *post hoc* test). (*H*) Western blot showing differential expression of cTnT isoforms between immature day 14 and mature day 30 iPSC-CMs, with graphs presented as mean ± S.E.M. (*n* = 3 independent experiments; one-way ANOVA followed by Tukey’s *post hoc* test).

The three iPSC lines were first validated for their pluripotency by staining against Nanog and Tra-1-81. Inter-line differences were not observed at the iPSC stage, as immunostaining revealed nuclear localization for Nanog and cell surface distribution for Tra-1-81, in all three iPSC lines (*Figure 1A* and see Supplementary material online, *Figure S1A*), thereby confirming their pluripotency. Additionally, a stem cell proteome profile array revealed a marked increase in pluripotency markers in all three iPSC lines (Oct-3/4 and SRY-box transcription factor 2 (SOX2)) as compared to markers expressed by differentiated cell populations (*Figure 1B* and see Supplementary material online, *Figure S1B*), thereby further validating their pluripotency. These iPSC lines were then subjected to cardiac differentiation, using a previously reported in-house protocol which is capable of generating relatively mature cardiac clusters by day 30 post-differentiation.^18^ Following differentiation, HCM-CM transcripts were sequenced to ensure that they retained the respective missense mutations (see Supplementary material online, *Figure S2A*).

Since cardiomyocyte enlargement is the hallmark of HCM, iPSC-CMs were first assessed for inter-line differences in cell size. When stained for sarcomeric α-actinin, immunofluorescence revealed HCM-1 and HCM-2 to be considerably larger in size in comparison to the control (Control 2393 ± 1187 µm^2^ vs. HCM-1 4206 ± 3341 µm^2^; *P* = 0.0031 vs. HCM-2 6722 ± 4472 µm^2^; *P* < 0.0001)) (*Figure 1C and D* and see Supplementary material online, *Figure S2B*). A counter-stain with DAPI also revealed HCM-1 and HCM-2 to comprise a higher percentage of multi-nucleate cells in comparison to the control (Control 5.76% ± 2.56% vs. HCM-1 22.5% ± 18.2%; *P* = 0.1889 vs. HCM-2 54.3% ± 31.0%; *P* < 0.0001; *Figure 1E*). Having observed increases in both cell size and multi-nucleate cells, we characterized the iPSC models further by determining the cellular distribution of NFAT4; a transcription factor which is retained in the cytosol, but capable of undergoing nuclear translocation under pathological stimuli, resulting in the activation of various maladaptive genes.^23^ When stained against NFAT4, immunofluorescence revealed minimal nuclear accumulation, with a predominant cytosolic distribution in the control, while an inverse staining pattern was observed in both HCM-1 and HCM-2 (*Figure 1F* and see Supplementary material online, *Figure S2C*). Since dephosphorylation of NFAT4 results in its nuclear accumulation,^24^ we assessed the level of phosphorylated NFAT4 at serine residues 165 (Ser-165) by western blot. Consistent with increased nuclear accumulation observed via immunofluorescence, western blot analysis revealed markedly decreased p-NFAT4 (Ser-165) in both HCM-1 and HCM-2 in comparison to the control (*Figure 1G*). Finally, to ensure that these observations were a result of disease pathophysiology and were not influenced by differential levels of cardiomyocyte maturity, western blotting was performed to assess the transition of cardiac troponin T isoforms, as their differential expression has been reported to coincide with various developmental stages.^25^ Consistent with our previous report,^18^ western blot analysis revealed a significant transition to the adult isoform in all 3 day 30 iPSC-CM lines in comparison to immature day 14 iPSC-CMs (*Figure 1H*), thereby confirming an advanced developmental stage. Collectively, the above findings confirmed that both HCM-1 and HCM-2 recapitulated the morphological characteristics of HCM.

### 3.2 HCM-CMs maintain contractile function but relaxation is impaired

**Figure 2:**
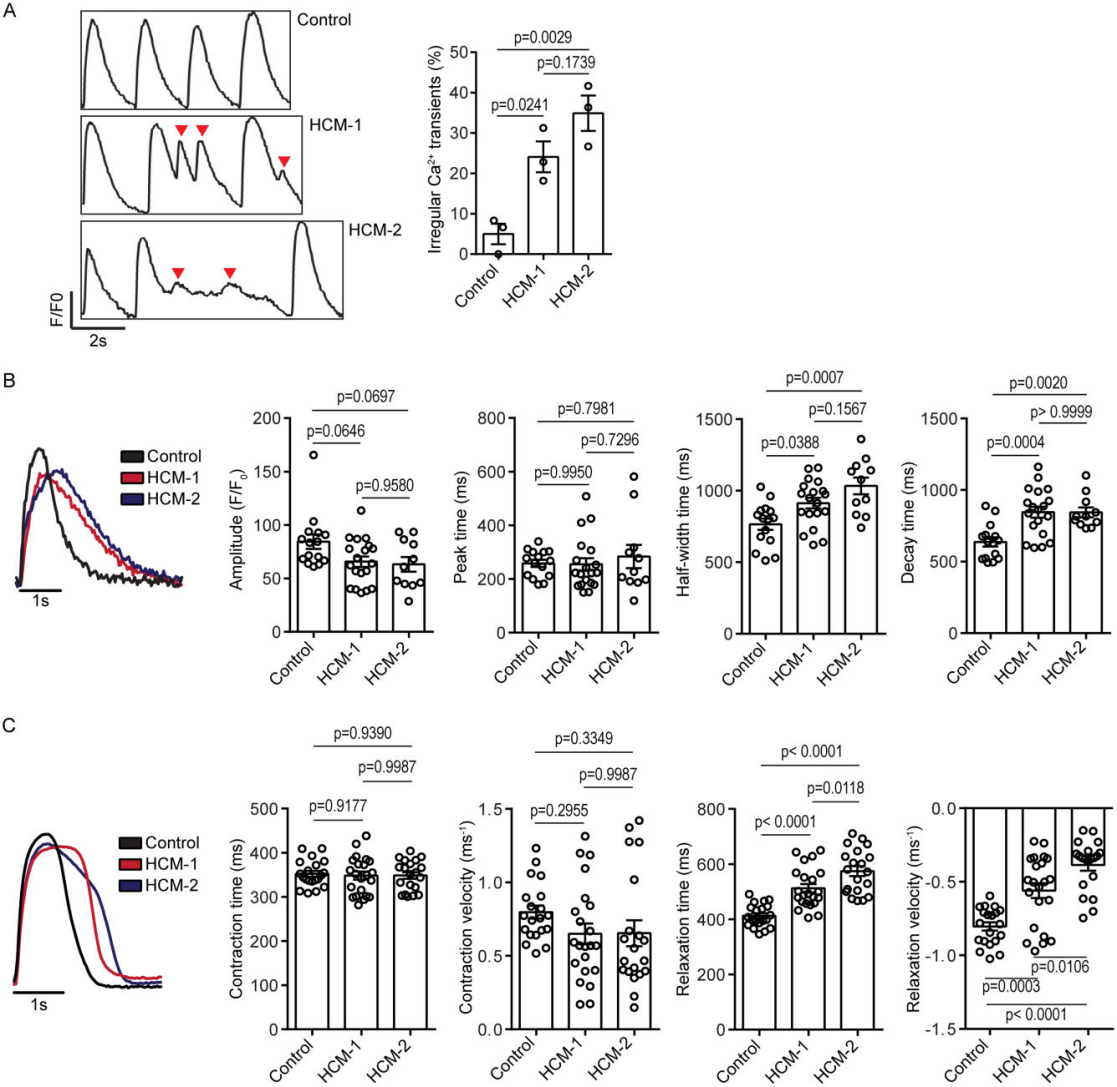
Functional characterization of HCM-CMs. (*A*) Representative calcium transient trace indicating irregular calcium transients (red arrowheads) in HCM-CMs, with graph presented as mean ± S.E.M. showing percentage of cells with irregular calcium transients (Control *n* = 35; HCM-1 *n* = 59; HCM-2 *n* = 38; one-way ANOVA followed by Tukey’s *post hoc* test; three independent experiments). (*B*) Representative calcium transient trace indicating prolonged half-width and decay times in HCM-CMs, with graphs presented as mean ± S.E.M. showing comparisons in calcium transient amplitude, peak time, half-width, time and decay time between control- and HCM-CMs (Control *n* = 15; HCM-1 *n* = 19; HCM-2 *n* = 11; one-way ANOVA followed by Tukey’s *post hoc* test; three independent experiments). (*C*) Representative contractile trace indicating prolonged relaxation times in HCM-CMs, with graphs presented as mean ± S.E.M. showing comparison in contraction time, contraction velocity, relaxation time, and relaxation velocity between control- and HCM-CMs (Control *n* = 20; HCM-1 *n* = 22; HCM-2 *n* = 20; one-way ANOVA followed by Tukey’s *post hoc* test; three independent experiments).

The most common cause of sudden cardiac death in patients with HCM is ventricular arrhythmia, and this has been attributed to abnormal calcium handling, and changes in myofilament calcium sensitivity.^4^ Therefore, we aimed to functionally validate HCM-1 and HCM-2 by first assessing calcium transients in comparison to the control. When paced (electrically stimulated) at 0.5 Hz, a significantly higher percentage of cells derived from both HCM-1 and HCM-2 exhibited irregular calcium transients (Control 5.00% ± 4.41% vs. HCM-1 24.1% ± 6.62%; *P* = 0.0241 vs. HCM-2 34.9% ± 7.61%; *P* = 0.0029; *Figure 2A*), suggesting a predisposition towards arrhythmic events. As these rhythm irregularities masked the finer details of the calcium transients, we also paced the cells at a much slower frequency of 0.25 Hz. When paced at this frequency, four parameters were compared between the control- and HCM-CMs, namely the calcium transient amplitude, peak time, half-width time, and decay time. Interestingly, calcium transient analysis indicated that while amplitude and peak time remained relatively similar, the half-width and decay times (Control 637 ± 125 ms vs. HCM-1 845 ± 168 ms; *P* = 0.0004 vs. HCM-2 844 ± 116 ms; *P* = 0.0020) were significantly prolonged in both HCM-1 and HCM-2, in comparison to the control (*Figure 2B*), suggesting impaired calcium signalling in the HCM-CMs.^26^

To investigate the effect of impaired calcium signalling on cardiomyocyte contractility, we measured the contraction time, contraction velocity, relaxation time, and relaxation velocity. The contractility measurements supported the calcium transient data by confirming the presence of impaired cardiomyocyte relaxation in both HCM-1 and HCM-2, as relaxation time was significantly prolonged (Control 413 ± 41.5 ms vs. HCM-1 512 ± 73.4 ms; *P* < 0.0001 vs. HCM-2 574 ± 80.9 ms; *P* < 0.0001), and relaxation velocity was significantly slower in comparison to control (*Figure 2C*). No significant inter-line differences were observed when assessing contraction parameters. Overall, the calcium transient and contractility data indicated that although HCM-1 and HCM-2 retain relatively normal contractile function, they possess an inherent relaxation defect. This is consistent with clinical findings of diastolic dysfunction, whereby HCM patients are reported to have delayed relaxation due to increased myocardial stiffness, which impairs diastolic filling.^2^^,^^3^

### 3.3 iPSC-CMs express functional MPO

**Figure 3:**
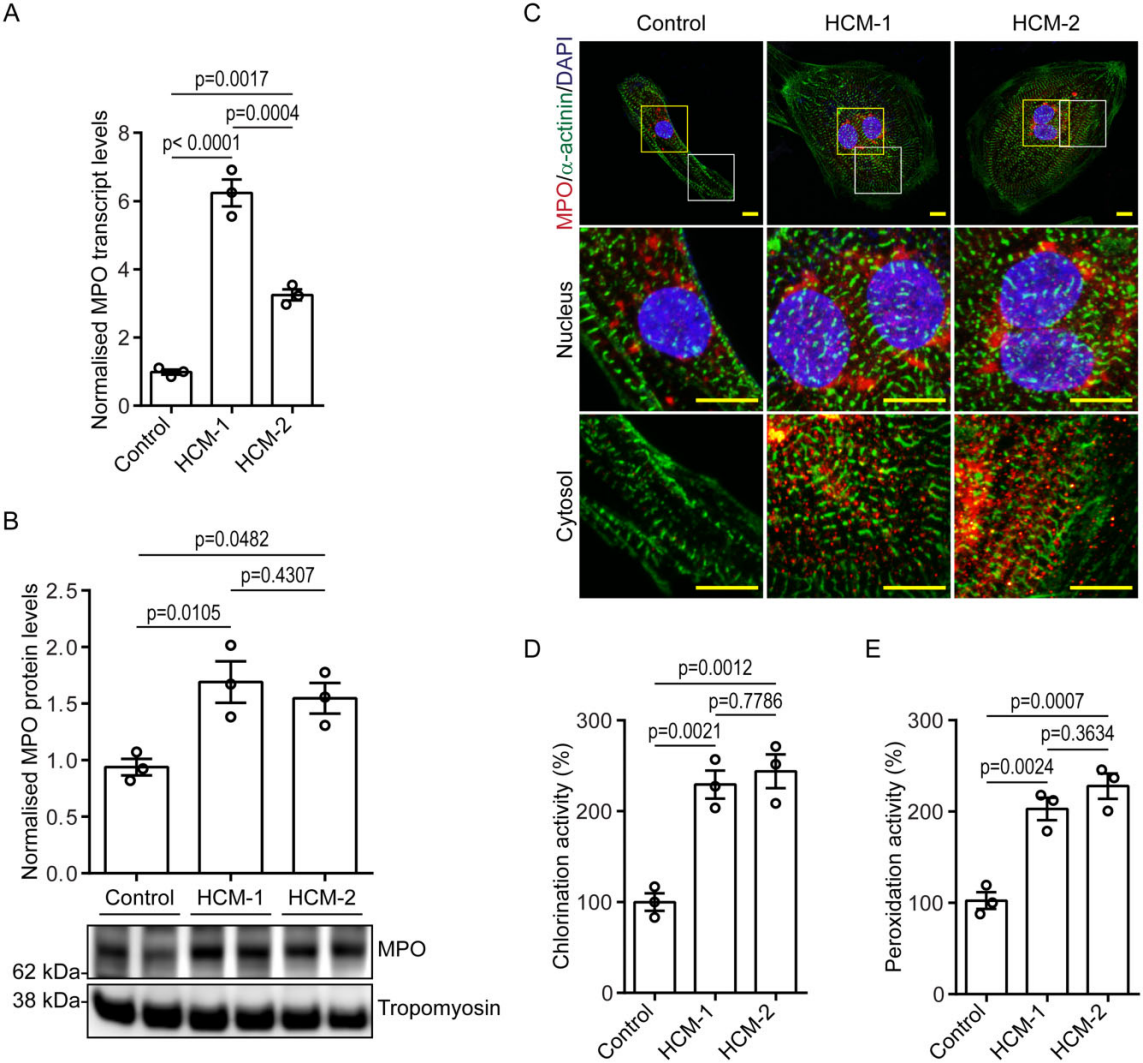
Validation of MPO expression in iPSC-CMs. (*A*) Graph presented as mean ± S.E.M. showing MPO transcript levels in control- and HCM-CMs (*n* = 3 independent experiments; one-way ANOVA followed by Tukey’s *post hoc* test). (*B*) Western blot showing MPO protein levels in control- and HCM-CMs, with graph presented as mean ± S.E.M. showing densitometry data normalized to tropomyosin (*n* = 3 independent experiments; one-way ANOVA followed by Tukey’s *post hoc* test). (*C*) Representative immunofluorescence pictographs of control- and HCM-CMs stained against MPO (red) and sarcomeric α-actinin (green), and counterstained with DAPI (blue). Yellow and white insets represent magnified nuclear and cytosolic regions, respectively. Scale bar: 10 µm. (*D* and *E*) Graphs presented as mean ± S.E.M. showing percentage of chlorination and peroxidation activity of MPO in control- and HCM-CMs (*n* = 3 independent experiments; one-way ANOVA followed by Tukey’s *post hoc* test).

Having characterized these HCM models on the cellular and functional levels, we aimed to identify a common cause for the observed relaxation defects, considering that both HCM-1 and HCM-2 harbour mutations in different sarcomere genes. Treatment of isolated human LV cardiomyocytes with exogenous MPO has been shown to induce contractile dysfunction,^27^ and since elevated MPO levels has been closely associated with worsening prognosis in CVDs,^10^ we hypothesized that MPO is endogenously expressed in iPSC-CMs and elevated under pathological conditions. Gene expression analysis revealed *MPO* transcripts to be present in control and were significantly up-regulated in both HCM-1 (approximately six-fold) and HCM-2 (approximately three-fold; *Figure 3A*). Sanger sequencing was performed on the amplified RT-PCR product, which confirmed it to be MPO (see Supplementary material online, *Figure S3A*), thereby ruling out the possible amplification of an artefact. Western blot analysis also revealed a similar trend, indicating that MPO was not only transcribed, but also translated in control iPSC-CMs, and even more so in HCM-CMs (*Figure 3B*). Although another peroxidase, peroxidasin (*PXDN* or *VPO*) has been implicated in angiotensin II-induced hypertrophy in rat myoblasts,^28^ our gene expression analysis indicated no significant inter-line differences in the levels of *PXDN* (see Supplementary material online, *Figure S3B*), thereby ruling it out as a probable cause of pathogenicity.

Since MPO is mainly stored in azurophilic granules of neutrophils, we were interested to determine its cellular localization in iPSC-CMs. When stained against MPO, immunofluorescence revealed a strong nuclear/perinuclear presence together, with a punctate distribution pattern throughout the cytosol in both HCM-1 and HCM-2 in comparison to the control (*Figure 3C* and see Supplementary material online, *Figure S3C*). This staining pattern was consistent with previous findings in neutrophils, whereby, the initiation of NETosis has been reported to induce the nuclear translocation of cytoplasmic granule proteins, including MPO.^29^

Having confirmed the expression and cellular presence of MPO in iPSC-CMs, we next assessed its functionality by assaying chlorination and peroxidation activity. The assays indicated significantly higher chlorination (*Figure 3D*) and peroxidation activity (*Figure 3E*) in HCM-1 and HCM-2 in comparison to the control, which was to be expected considering that HCM-CMs expressed MPO at significantly higher levels than the control (*Figure 3A and B*). Apart from revealing that MPO is up-regulated under pathological conditions, these findings also indicate that the MPO present within the iPSC-CMs is functional.

### 3.4 MPO is expressed in adult cardiomyocytes and is elevated in response to ROS

**Figure 4:**
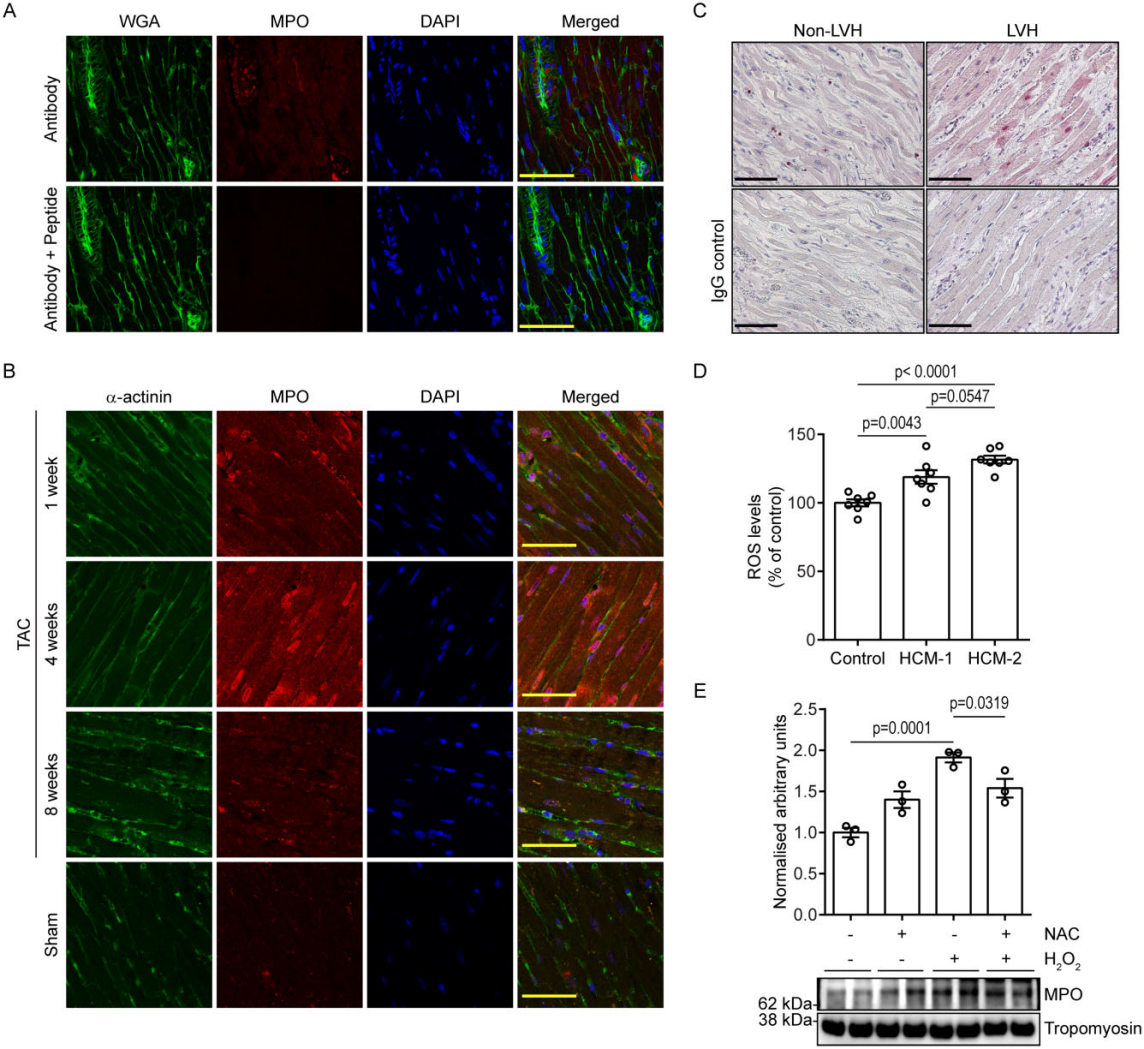
Validation of MPO expression in adult cardiac tissue. (*A*) Representative immunofluorescence pictographs of mouse cardiac tissue stained against MPO and wheat germ agglutinin (WGA), and counterstained with DAPI in the presence or absence of recombinant MPO protein fragment. Scale bar: 50 µm. (*B*) Representative immunofluorescence pictographs of mouse cardiac tissue at 1, 4, and 8 weeks post-TAC stained against MPO and sarcomeric α-actinin, and counterstained with DAPI. Scale bar: 50 µm. (*C*) Representative immunohistochemistry pictographs of human LVH and non-LVH cardiac tissue stained against MPO (pink) with corresponding IgG controls taken from the same area. Scale bar: 100 µm. (*D*) Graph presented as mean ± S.E.M. showing ROS levels in control- and HCM-CMs (one-way ANOVA followed by Tukey’s *post hoc* test; three independent experiments totalling seven wells). (*E*) Western blot showing MPO levels in control-CMs following treatment with hydrogen peroxide and NAC, with graph presented as mean ± S.E.M. showing densitometry data normalized to tropomyosin (*n* = 3 independent experiments; one-way ANOVA followed by Tukey’s *post hoc* test).

Having identified endogenous cardiomyocyte MPO in iPSC-CMs, we next assessed MPO expression in adult cardiac tissue through a series of immunofluorescence and immunohistochemical studies. When mouse cardiac tissue was stained against MPO, immunofluorescence using high-resolution microscopy revealed positive staining in endothelial cells^12^ and punctate cytosolic expression in cardiomyocytes (*Figure 4A*). To rule out detection of possible artefacts, we tested the specificity of the antibody by co-incubation with a recombinant MPO protein fragment (Abcam; ab158915). Following co-incubation with antibody and peptide, immunofluorescence revealed a complete lack of signal confirming the specificity of the antibody and its affinity towards MPO (*Figure 4A*).

Since we had confirmed that MPO is expressed at basal levels in mouse cardiac tissue, we were interested to determine if MPO is up-regulated under conditions of stress (similar to HCM-CMs) and for this we assessed the presence of MPO in a murine model of pressure overload-induced LVH. When stained against MPO, immunofluorescence revealed a marked increase at 4 weeks post-TAC in comparison to 1 and 8 weeks post-TAC (*Figure 4B*). Despite differential expression at various time-points, more MPO was detected in cardiac tissue from TAC mice than in tissue from sham mice. Interestingly, in addition to the punctate and diffused cytosolic distribution pattern that was observed at both 1- and 8-week timepoints, a strong nuclear/perinuclear presence was observed at 4 weeks post-TAC. Consistently, immunohistochemical analysis of human cardiac tissue from LVH subjects revealed strong nuclear and diffused cytosolic MPO expression in comparison to tissue obtained from non-LVH subjects (*Figure 4C*). These findings confirmed that MPO is expressed in cardiomyocytes from adult heart tissue, and is up-regulated under conditions of stress.

Considering that MPO is up-regulated in conditions of stress, we determined why MPO levels were increased in HCM-CMs. Increased ROS production is well known to be associated with both acquired and genetic forms of cardiac hypertrophy.^30^^,^^31^ In support, evaluation of dichlorodihydrofluorescein levels revealed a significant increase in ROS in both HCM-1 and HCM-2 in comparison to the control (*Figure 4D*). We hypothesized that this increase in ROS could be a determinant of MPO elevation in HCM-CMs. To test this hypothesis, control-CMs were treated with hydrogen peroxide (100 nM) for 24 h (to induced ROS formation) in the presence or absence of ROS scavenger, N-acetylcysteine (NAC; 100 µM) and MPO levels were assessed by western blot. Interestingly, western blot analysis revealed a significant increase in MPO expression following hydrogen peroxide treatment which was suppressed by NAC, thereby confirming ROS accumulation to be a determinant of MPO elevation (*Figure 4E*). In support of our observations, a previous study in rats reported increased ROS production to occur early in the development of pressure overload-induced heart failure, peaking at the onset of diastolic dysfunction^32^ which may explain the transient increase in MPO expression observed during the early stages of our TAC model.

### 3.5 MPO inhibition alleviates the relaxation defect in HCM-CMs

**Figure 5:**
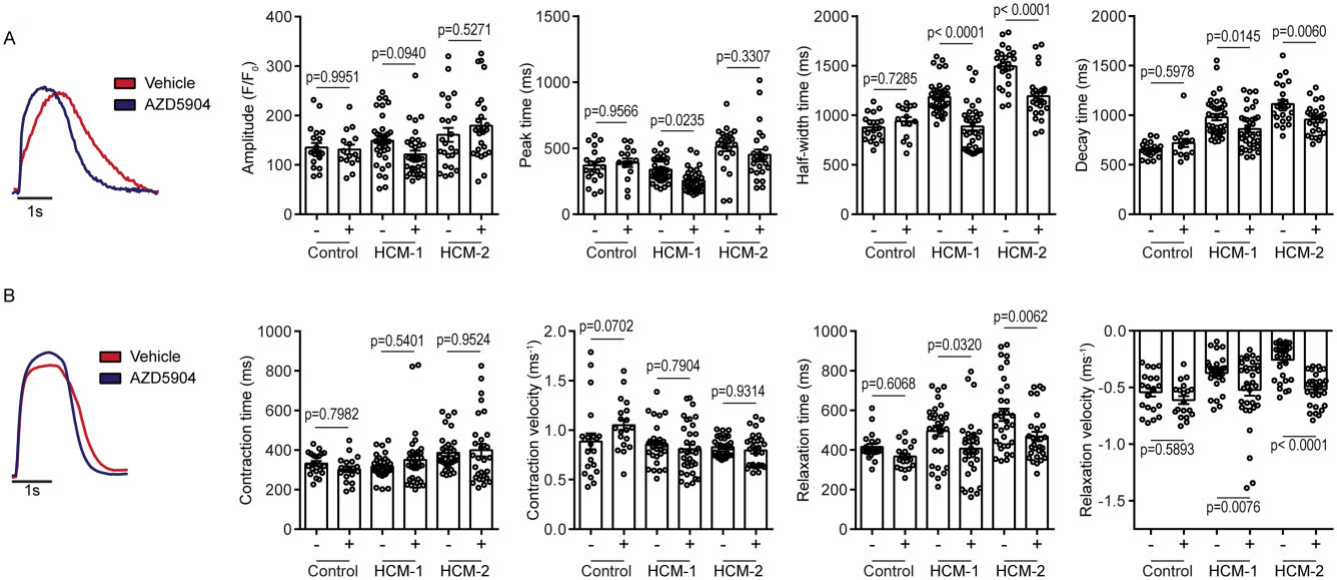
Evaluation of MPO inhibition on HCM-CM function. (*A*) Representative calcium transient trace of HCM-2 indicating reduction in half-width and decay times post-AZD5904 treatment. Graphs presented as mean ± S.E.M. showing comparisons in calcium transient amplitude, peak time, half-width time and decay time pre- and post-AZD5904 treatment (vehicle-treated Control *n* = 20; AZD5904-treated Control *n* = 15; vehicle-treated HCM-1 *n* = 38; AZD5904-treated HCM-1 *n* = 34; vehicle-treated HCM-2 *n* = 24; AZD5904-treated HCM-2 *n* = 26; one-way ANOVA followed by Sidak’s *post hoc* test; three independent experiments). (*B*) Representative contractile trace of HCM-2 indicating reduction in relaxation times post-AZD5904 treatment. Graphs presented as mean ± S.E.M. showing comparison in contraction time, contraction velocity, relaxation time and relaxation velocity pre- and post-AZD5904 treatment (vehicle-treated Control *n* = 21; AZD5904-treated Control *n* = 19; vehicle-treated HCM-1 *n* = 30; AZD5904-treated HCM-1 *n* = 34; vehicle-treated HCM-2 *n* = 33; AZD5904-treated HCM-2 *n* = 29; one-way ANOVA followed by Sidak’s *post hoc* test; three independent experiments).

Considering that both HCM-1 and HCM-2 express functional MPO at significantly higher levels than in control, we hypothesized this to be the main contributing factor for the observed cardiomyocyte relaxation defect. We therefore investigated whether MPO inhibition could alleviate the relaxation defects observed in HCM-CMs and for this purpose, we used the potent (IC_50_ = 0.2 µM) and selective (25-fold towards TPO, and at least 70-fold towards a panel of other enzymes, ion channels and receptors investigated *in vitro*) MPO inhibitor AZD5904^13^ in this study. To identify a suitable dose, we assessed the ability of the compound to reduce chlorination activity of MPO in a dose-dependent manner. In comparison to the vehicle-treated group, at a concentration of 10 µM, AZD5904 was capable of reducing the chlorination activity by more than 90% and was therefore considered for all further studies (see Supplementary material online, *Figure S4*). Having identified a suitable concentration, we then investigated whether chronic treatment (1 week) with AZD5904 exerted any (long-term) beneficial effects on calcium transients and contractility of HCM-CMs. Calcium transient analysis indicated that AZD5904 treatment did not have a significant effect on amplitude; however, the peak time for HCM-1 (but not HCM-2) was significantly reduced (*Figure 5A*). Interestingly, the half-width and decay times (HCM-1 vehicle 980 ± 195 ms vs. treatment 861 ± 201 ms; *P* = 0.0145; HCM-2 vehicle 1113 ± 200 ms vs. treatment 956 ± 154 ms; *P* = 0.0060) were significantly reduced in both HCM-1 and HCM-2 post-treatment (*Figure 5A*), suggesting alleviation of the calcium signalling defect observed previously. To validate this, we assessed the contractility of HCM-CMs post-treatment. The contractility measurements supported alleviation of the cardiomyocyte relaxation defect post-treatment, as both HCM-1 and HCM-2 exhibited significantly shorter relaxation times (HCM-1 vehicle 496 ± 144 ms vs. treatment 407 ± 152 ms; *P* = 0.0320: HCM-2 vehicle 578 ± 180 ms vs. treatment 468 ± 127 ms; *P* = 0.0062) and significantly faster relaxation velocities in comparison to vehicle-treated counterparts (*Figure 5B*). AZD5904 treatment had no significant effect on contractile parameters. As expected AZD5904 had no effect on calcium transients and contractility in control-CMs; however, these findings collectively support that MPO inhibition improved contractility in HCM-CMs, particularly by alleviating the relaxation defect.

### 3.6 MPO affects MYBPC3 phosphorylation status

Having observed the beneficial effects of MPO inhibition on improving calcium handling and contractility in HCM-CMs, our next aim was to identify a potential mechanism by which MPO adversely affected these parameters. Since calcium transient analysis indicated that both HCM-1 and HCM-2 exhibited prolonged half-width and decay times in comparison to the control (*Figure 2B*), we initially speculated the probability of decreased sarco/endoplasmic reticulum Ca(2+)-ATPase (SERCA) expression in HCM-CMs, which is reported to hamper calcium re-uptake into the sarcoplasmic reticulum, thereby impeding relaxation kinetics.^33^ However, gene expression analysis revealed SERCA (*ATP2A2*) transcripts to be up-regulated in both HCM1 and HCM-2 in comparison to the control (see Supplementary material online, *Figure S5*), suggesting that the observed relaxation defect was likely due to an alternative mechanism.

**Figure 6:**
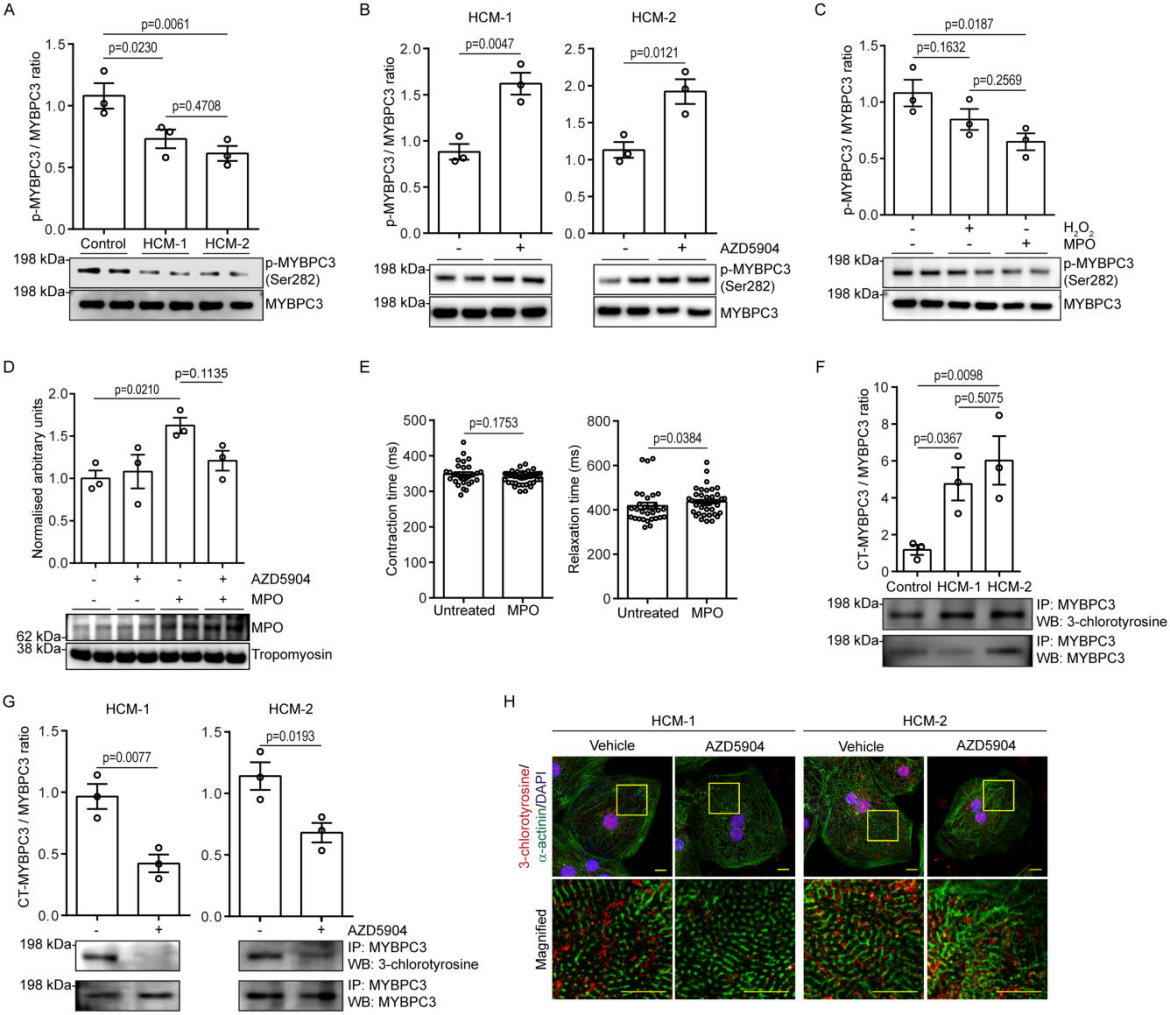
Determination of molecular mechanism by which MPO induces relaxation defect in HCM-CMs. (*A*) Western blot showing phosphorylated MYBPC3 levels in control- and HCM-CMs, with graph presented as mean ± S.E.M. showing densitometry data normalized to total MYBPC3 (*n* = 3 independent experiments; one-way ANOVA followed by Tukey’s *post hoc* test). (*B*) Western blots showing phosphorylated MYBPC3 levels pre- and post-AZD5904 treatment in HCM-1 and HCM-2, with graphs presented as mean ± S.E.M. showing densitometry data normalized to total MYBPC3 (*n* = 3 independent experiments; Unpaired *t*-test with Welch’s correction). (*C*) Western blot showing phosphorylated MYBPC3 levels in control-CMs following treatment with hydrogen peroxide and exogenous MPO, with graph presented as mean ± S.E.M. showing densitometry data normalized to total MYBPC3 (*n* = 3 independent experiments; one-way ANOVA followed by Tukey’s *post hoc* test). (*D*) Western blot showing MPO levels in control-CMs following treatment with exogenous MPO and AZD5904, with graph presented as mean ± S.E.M. showing densitometry data normalized to tropomyosin (*n* = 3 independent experiments; one-way ANOVA followed by Tukey’s *post hoc* test). (*E*) Graphs presented as mean ± S.E.M. showing comparison in contraction time and relaxation time in control-CMs pre- and post-MPO treatment (vehicle *n* = 30; MPO *n* = 42; Unpaired *t*-test with Welch’s correction for contraction time; Mann–Whitney test for relaxation time; three independent experiments). (*F*) Immunoprecipitation showing 3-chlorotyrosine-modified MYBPC3 levels in control- and HCM-CMs, following pull-down with total MYBPC3. Graph presented as mean ± S.E.M. showing densitometry data normalized to total pull-down MYBPC3 (*n* = 3 independent experiments; one-way ANOVA followed by Tukey’s *post hoc* test). (*G*) Immunoprecipitation showing 3-chlorotyrosine-modified MYBPC3 levels pre- and post-AZD5904 treatment in HCM-1 and HCM-2, following pull-down with total MYBPC3. Graphs presented as mean ± S.E.M. showing densitometry data normalized to total pull-down MYBPC3 (*n* = 3 independent experiments; Unpaired *t*-test with Welch’s correction). (*H*) Representative immunofluorescence pictographs of HCM-1 and HCM-2 pre- and post-AZD5904 treatment stained against 3-chlorotyrosine (red) and sarcomeric α-actinin (green), and counterstained with DAPI (blue). Yellow inset represents magnified region. Scale bar: 10 µm.

Since both HCM-1 and HCM-2 had prolonged relaxation times and slower relaxation velocities, we also speculated potential alterations in the phosphorylation status of sarcomeric proteins, in particular MYBPC3, which is reported to be a critical mediator of diastolic function in HCM.^34^^,^^35^ We found that the phosphorylation status of serine residue 282 (Ser-282) on MYBCP3, was significantly decreased in both HCM-1 (∼1.4-fold) and HCM-2 (∼1.6-fold) in comparison to the control (*Figure 6A*). Interestingly, following treatment with AZD5904, western blot analysis revealed a significant increase in p-MYBPC3 (Ser-282) in both HCM-1 (∼1.6-fold) and HCM-2 (∼1.9-fold; *Figure 6B*), suggesting MPO to be responsible for the reduced phosphorylation status. To further validate that MPO was indeed responsible for the reduced levels of p-MYBPC3 (Ser-282) observed in HCM-CMs, we treated control cells with exogenous MPO (100 ng/ml) for 48 h. In comparison to the vehicle-treated group, western blot analysis revealed a significant decrease in p-MYBPC3 (Ser-282) levels post-MPO treatment (*Figure 6C*). Hydrogen peroxide, which was used as an internal control had no significant effect on p-MYBPC3 (Ser-282) thereby, confirming that the reduced phosphorylation status observed was a direct effect of MPO. Since exogenous MPO alone was sufficient to influence p-MYBPC3 (Ser-282) levels, we hypothesized that this was likely mediated by a positive feedback mechanism as MPO has been reported to be involved in chromatin decondensation independent of its enzyme activity.^36^ To test this hypothesis, control-CMs were treated with exogenous MPO (100 ng/ml) in the presence or absence of AZD5904 and intracellular MPO levels were assessed by western blot. Supporting our hypothesis, western blot analysis revealed a marked increase in intracellular levels following MPO treatment that could not be supressed by AZD5904 (*Figure 6D*). Finally, we have shown that acute treatment (48 h) of control-CMs with exogenous MPO resulted in impaired relaxation (*Figure 6E* and see Supplementary material online, *Figure S6A*).

### 3.7 MYBPC3 undergoes 3-chlorotyrosine modification in HCM-CMs

A specific downstream product of MPO activity is 3-chlorotyrosine. Since MPO inhibition increased p-MYBPC3 (Ser-282) levels in HCM-CMs (*Figure 6B*) and conversely, treatment with exogenous MPO decreased p-MYBPC3 (Ser-282) in control-CMs (*Figure 6C*), we hypothesized that MPO interfered with MYBPC3 phosphorylation via the generation of 3-chlorotyrosine, as modified tyrosine residues have been previously shown to alter the phosphorylation status of various proteins.^37^ To test this, an immunoprecipitation assay was performed which revealed both HCM-1 (∼4.7-fold) and HCM-2 (approximately six-fold) to comprise significant amounts of 3-chlorotyrosine-modified MYBPC3 protein in comparison to the control (*Figure 6F*), which could be significantly depleted upon treatment with AZD5904 (*Figure 6G*). This was further supported by immunofluorescence analysis, which suggested an overall reduction in the intra-cellular levels of 3-chlorotyrosine post-AZD5904 treatment (*Figure 6H* and see Supplementary material online, *Figure S6B*). Collectively, these findings imply that the relaxation defect observed in HCM-CMs was primarily due to an MPO-mediated modification of the MYBPC3 protein, which resulted in a reduced phosphorylation status.

## 4. Discussion

In this study, we demonstrate for the first time the presence of functional MPO in iPSC-CMs. We found that endogenous MPO levels are up-regulated in HCM-CMs, where it increases 3-chlorotyrosine-modified MYBPC3 protein levels, and reduces phosphorylation of MYBPC3, which in turn impairs cardiomyocyte relaxation kinetics in HCM-CMs. Importantly, these effects were reversed by treatment with AZD5904, an MPO inhibitor which has been tested in Phase I clinical trials.

A major novel finding of our study is demonstrating for the first time that iPSC-CMs express MPO which is up-regulated in HCM-CMs. Although a number of previous clinical studies have shown an association of elevated plasma MPO levels with poor prognosis in CVDs,^10^ studies investigating the direct effect of MPO on heart contractile function or even the possibility of MPO being expressed in cardiomyocytes *per se* are limited. In a previous study, it was shown in the murine heart that acute myocardial ischaemia and reperfusion increased myocardial expression of MPO, but this was attributed to infiltrating leucocytes.^38^ In our study, to exclude the possibility of cardiomyocyte MPO signal arising from other cell types present within the cardiac clusters, immunofluorescence analysis of dissociated single-cell iPSC-CMs supported the expression of cardiomyocyte MPO. Similarly, high-resolution microscopy and antibody blocking studies confirmed the presence of cardiomyocyte MPO in adult mouse and human cardiac tissue that was found to be elevated in the setting of LVH. Although the detection of MPO within cardiomyocytes could be attributable to non-professional phagocytosis,^39^ the existence of cardiomyocyte MPO is consistent with a recent large-scale single nuclei RNA sequencing study that confirmed its presence in atrial and ventricular cardiomyocyte populations derived from healthy human hearts.^40^ Intracellular MPO is critical for the antimicrobial activity of neutrophils; however, due to its ability to generate reactive species and chemically modify various cellular components, dysregulated MPO release due to oxidative stress or inflammation can lead to extensive tissue damage as seen in numerous diseases.^7^^,^^9^ Cardiomyocyte MPO may also exert a similar effect, independent of neutrophil activation, as our immunoprecipitation data has revealed 3-chlorotyrosine modification of the MYBPC3 protein, which subsequently resulted in impaired relaxation kinetics in HCM-CMs.

Of the 1400 mutations that have been associated with HCM, those in MYH7 and MYBPC3 account for ∼70% of cases.^1^ MYBPC3 in particular is reported to be a critical mediator of diastolic function, with its level of phosphorylation modulating cross-bridge detachment rate in relation to attachment rate.^41^ In support of this finding, LV myectomy samples obtained from HCM patients have been demonstrated to show an ∼60% decrease in the levels of phosphorylation of MYBPC3, compared with control tissue.^42^*In vivo* studies have reported phosphorylation of MYBPC3 to occur at three serine sites (Ser-273, Ser-282, and Ser-302),^43^ and while Ser-282 phosphorylation mediates the subsequent phosphorylation of Ser-302, its ablation results in impaired diastolic function, with no significant impact on contractility.^44^ Consistent with these findings, our western blot and contractility data also support a positive correlation between reduced p-MYBPC3 (Ser-282) levels, and relaxation defects in HCM-CMs. In a recent study, iPSC-CMs derived from HCM patients were reported to exhibit a diastolic dysfunction phenotype, which could be alleviated by partial blockade of calcium or late sodium currents, suggesting that abnormal calcium handling plays an important role in disease pathogenesis.^26^ Similarly, our HCM-CMs also exhibited abnormal calcium transients, in particular a prolonged half-width and decay time, suggestive of impaired calcium re-uptake. However, the elevated *ATP2A2* levels in our HCM-CMs and the ability to alleviate the relaxation defect via increasing MYBPC3 phosphorylation status would suggest that the relaxation defect observed was primarily due to defective sarcomere mechanics, with abnormal calcium handling a secondary effect of disease pathophysiology.

The key finding of this study in terms of translational and therapeutic importance was that MPO inhibition alleviated the relaxation defect observed in HCM-CMs, by augmenting p-MYBPC3 (Ser-282) levels. 3-chlorotyrosine is one of the major products of MPO-catalysed reactions and is reported to alter the function of apolipoprotein A-I by depleting ABCA1-dependent cholesterol efflux activity.^6^ Comparatively, our immunoprecipitation data revealed HCM-CMs to contain significantly higher levels of 3-chlorotyrosine-modified MYBPC3, which upon depletion, resulted in an increased phosphorylation status of MYBPC3 that improved relaxation kinetics. X-ray crystallography studies have revealed that modifications on tyrosine residues can alter protein structure and function by inducing conformational changes and causing steric hindrance.^45^ Therefore, we speculate that the 3-chlorotyrosine modification of MYBPC3 induced by MPO in HCM-CMs, prevents the phosphorylation of MYBPC3 by a conformational change and steric hindrance. Considering that MYBPC3 is closely associated with essentially all the components of the sarcomere; including actin, myosin, and titin, it is reasonable to assume that even slight alterations to this protein could have profound effects on contractility. Consistent with this finding, treatment with exogenous MPO has been reported to impair contractility in isolated LV human cardiomyocytes via chemical modification of actin and MYBPC3 but, with no changes to titin.^27^ Similarly, we found that acute treatment of control-CMs with exogenous MPO resulted in impaired relaxation with reduced MYBPC3 phosphorylation.

Although a potential limitation of this study is the lack of isogenic controls, which is specifically used to determine genotype to phenotype relationships, our findings do support a common mechanism, independent of gene mutation, through which increased MPO levels perturb MYBPC3 phosphorylation status, which in turn impairs relaxation in two different HCM iPSC lines. Although we have shown an increase in ROS to be a key determinant of MPO elevation, other mechanisms that contribute to its up-regulation should also be considered as polymorphisms within the *MPO* gene that potentially alter expression levels has been reported to be associated with coronary artery disease.^46^

In summary, by inhibiting MPO, which was found to be elevated in HCM-CMs, we were able to improve contractility, by alleviating the relaxation defect. MPO inhibition, may therefore provide a novel therapy for improving diastolic function in HCM patients, a treatment strategy which can be easily tested given that MPO inhibitors are already available for clinical testing. Furthermore, MPO inhibition may also be beneficial in other cardiac conditions characterized by impaired diastolic function such as LVH due to arterial hypertension and heart failure with preserved ejection function.

## Supplementary material

Supplementary material is available at *Cardiovascular Research* online.

## Authors’ contributions

C.J.A.R., W.S., and D.J.H. were involved in conception, design of the work, analysis and interpretation of data. K.P.M.M.J., J.C., Y.H.L., S.H.R., E.A.L., H.C.T., and P.W. were involved in acquisition of data. S.S., L.M.G., M.K.B.J., E.M., K.R.M., R.V.B., and R.F.D. were involved in analysis and interpretation of data. C.J.A.R. and D.J.H. drafted the article. All authors approve the submitted version.

## Supplementary Material

cvab077_Supplementary_Data

## Notes

Translational perspective There are currently no specific therapies for improving diastolic function in patients with hypertrophic cardiomyopathy (HCM). We show for the first time that myeloperoxidase (MPO) is present in and is up-regulated in cardiomyocytes derived from human induced pluripotent stem cells obtained from HCM patients, where it impairs cardiomyocyte relaxation by reducing phosphorylation of cardiac myosin binding protein-C (MYBPC3). Treatment with the MPO inhibitor, AZD5904, restored MYBPC3 phosphorylation and alleviated the relaxation defect, demonstrating cardiomyocyte MPO to be a novel therapeutic target for improving diastolic function in HCM, a treatment strategy which can be evaluated in HCM patients given that MPO inhibitors are already available for clinical testing.
