# Corrigendum to: Inhibiting cardiac myeloperoxidase alleviates the relaxation defect in hypertrophic cardiomyocytes

**DOI:** 10.1093/cvr/cvab130

**Published:** 2021-04-30

*Cardiovasc Res.*, cvab077, https://doi.org/10.1093/cvr/cvab077

The authors have requested that Ralph Knöll be removed from the acknowledgments section of the paper and be added as a co-author.

This change does not affect the overall scientific message of the paper.

