# Supplementary material for: Inhibiting cardiac myeloperoxidase alleviates the relaxation defect in hypertrophic cardiomyocytes
Source: Cardiovasc Res. 2021 Mar 11;118(2):517–30. doi: 10.1093/cvr/cvab077 (PMC8803077; doi:10.1093/cvr/cvab077)
Supplement: cvab077_Supplementary_Data [file cvab077_supplementary_data.pdf]

Inhibiting cardiac myeloperoxidase alleviates the relaxation defect in hypertrophic cardiomyocytes

Chrishan J.A. Ramachandra, Myu Mai Ja Kp, Jasper Chua, Sauri Hernandez-Resendiz, Elisa A. Liehn, Li-Ming Gan, Erik Michaëlsson, Malin K.B. Jonsson, Katarina Ryden-Markinhuhta, Ratan V. Bhat, Regina Fritsche-Danielson, Ying-Hsi Lin, Sakthivel Sadayappan, Hak Chiaw Tang, Philip Wong, Winston Shim[#] and Derek J. Hausenloy[#]

[#]Joint senior authors

Supplementary Data

Supplementary figure 1

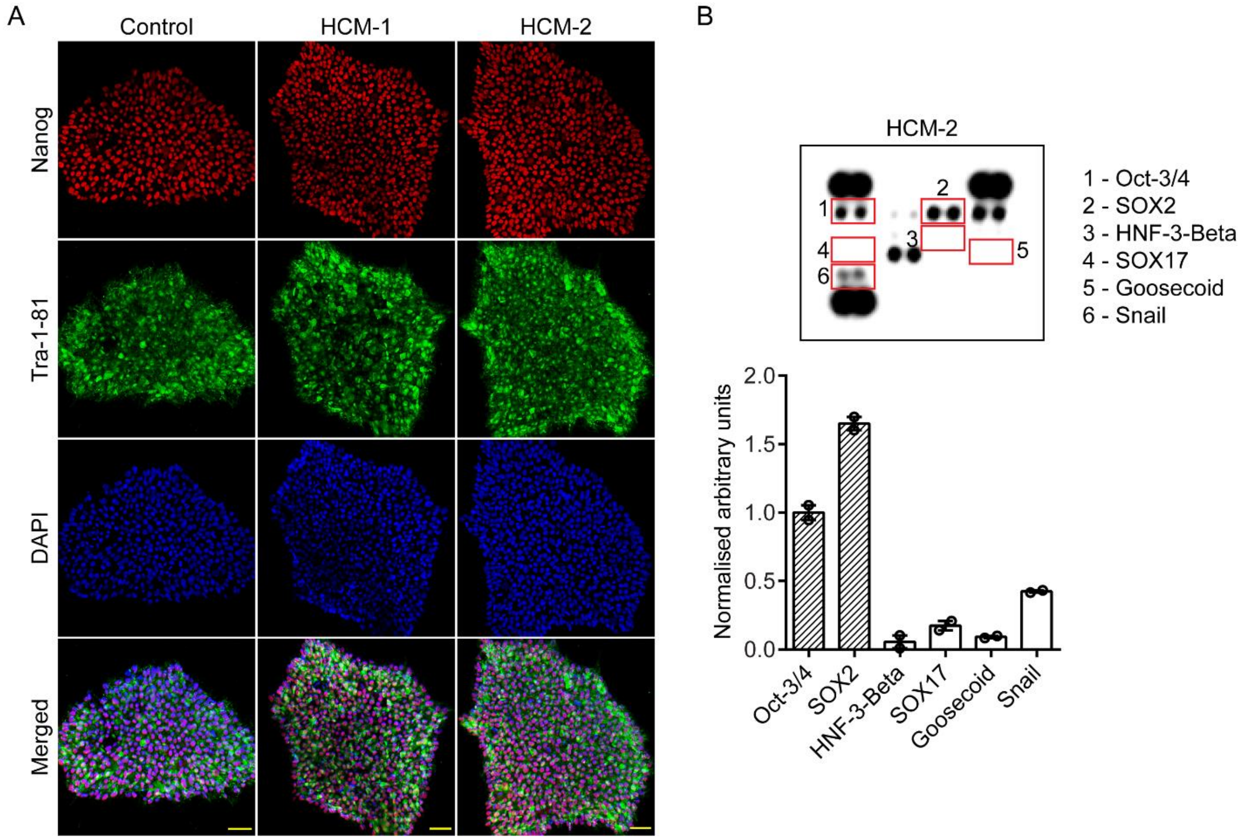

Supplementary figure 1: (**A**) Single channel and merged pictographs of control- and HCM-iPSCs stained against pluripotency antigens; Nanog and Tra-1-81 and counterstained with DAPI. Scale bar: 50µm. (**B**) Proteome profiler array showing expression of pluripotent (shaded bars) and non-pluripotent markers (clear bars) in HCM-2 iPSCs with graph presented as mean ± s.e.m. showing densitometry data normalised to internal control (n=2 independent experiments).

Supplementary figure 2

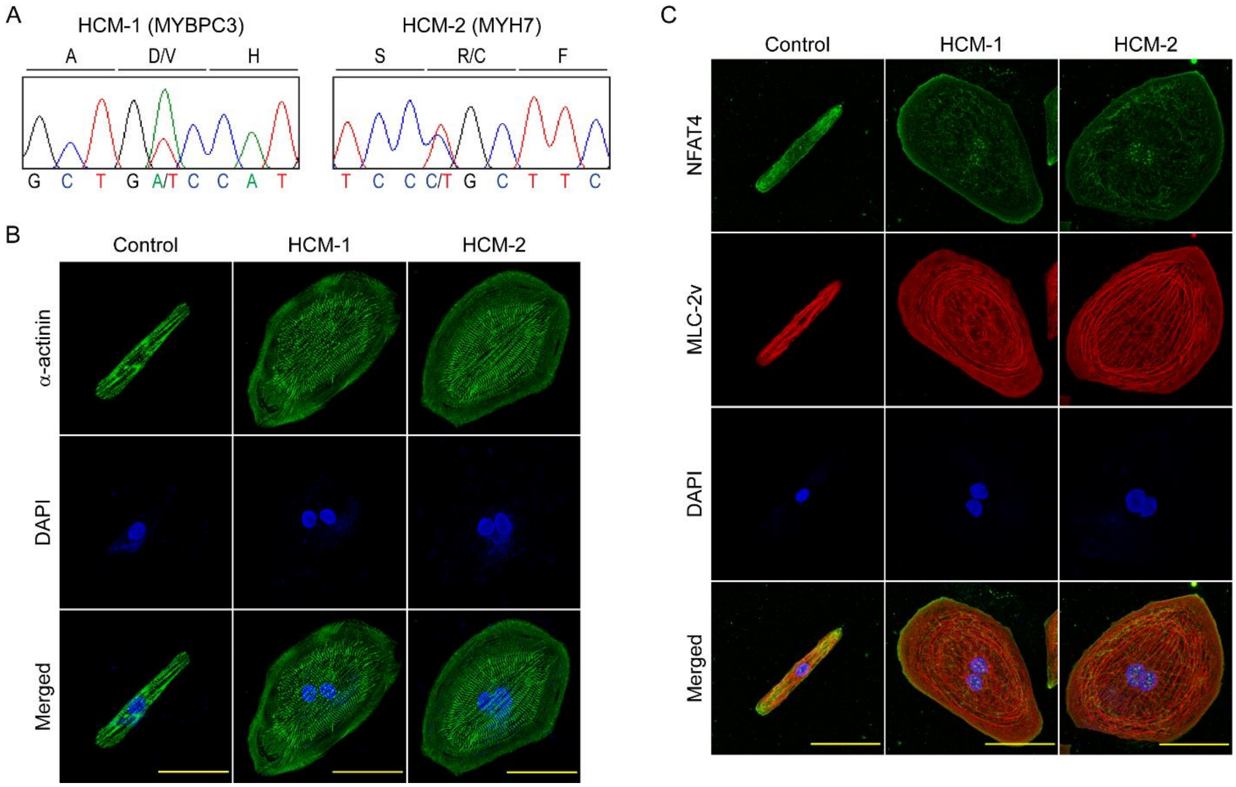

Supplementary figure 2: (**A**) Sanger sequencing confirmed the presence of a heterozygous missense mutation in the sarcomere transcripts of HCM-CMs. (**B**) Single channel and merged pictographs of control- and HCM-CMs stained against sarcomeric α-actinin and counterstained with DAPI. Scale bar: 50µm. (**C**) Single channel and merged pictographs of control- and HCM-CMs stained against NFAT4 and MLC-2v and counterstained with DAPI. Scale bar: 50µm.

Supplementary figure 3

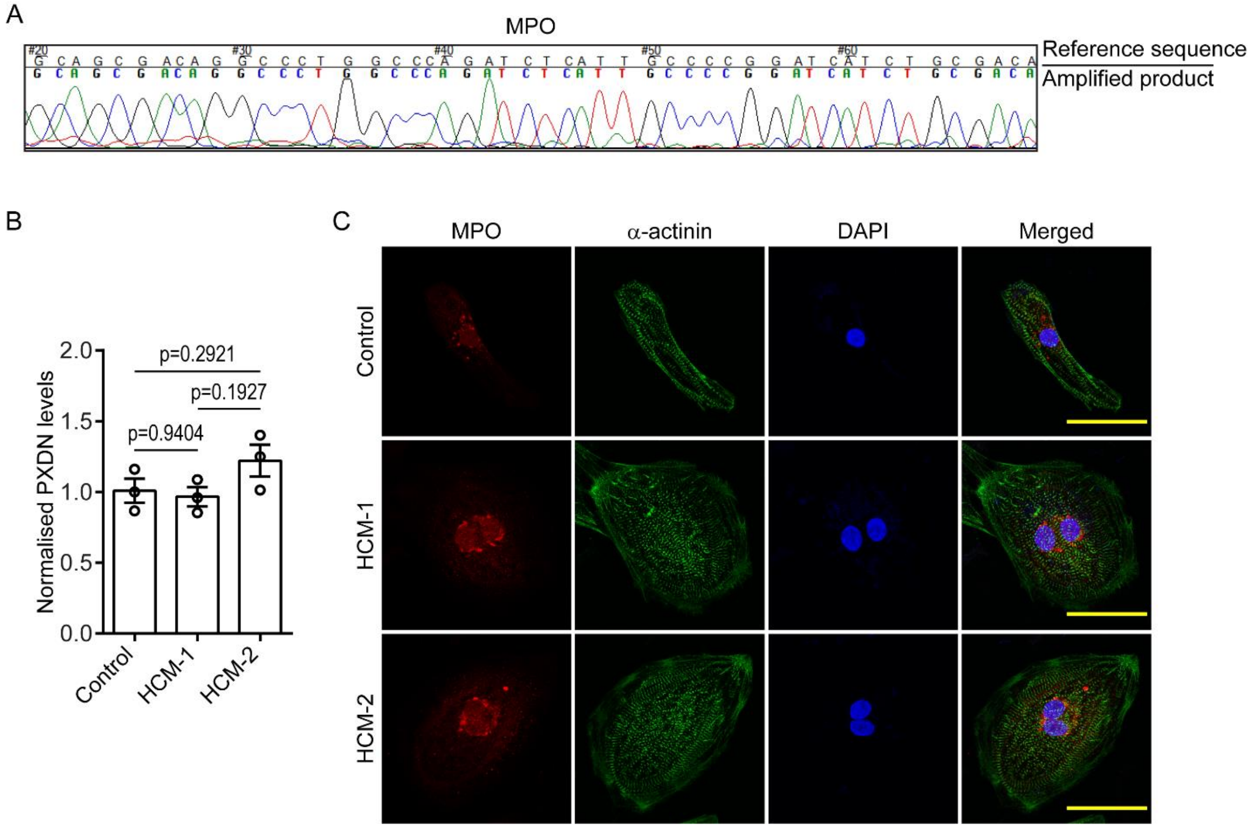

Supplementary figure 3: (**A**) Sanger sequencing confirmed that the amplified transcript from iPSC-CMs is MPO. (**B**) Graph presented as mean ± s.e.m. showing *PXDN* transcript levels in control- and HCM-CMs (n=3 independent experiments; One-way ANOVA followed by Tukey's post-hoc test). (**C**) Single channel and merged pictographs of control- and HCM-CMs stained against MPO and sarcomeric α-actinin and counterstained with DAPI. Scale bar: 50µm.

Supplementary figure 4

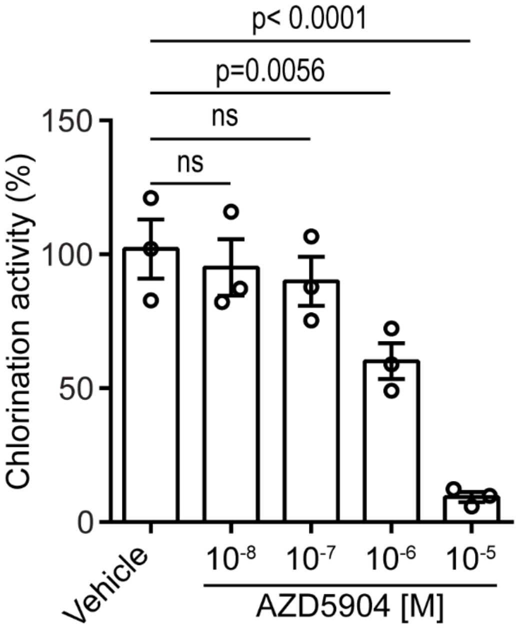

Supplementary figure 4 Graph presented as mean ± s.e.m. showing percentage of chlorination activity post-AZD5904 treatment at varying concentrations (n=3 independent experiments; One-way ANOVA followed by Dunnett's post-hoc test).

Supplementary figure 5

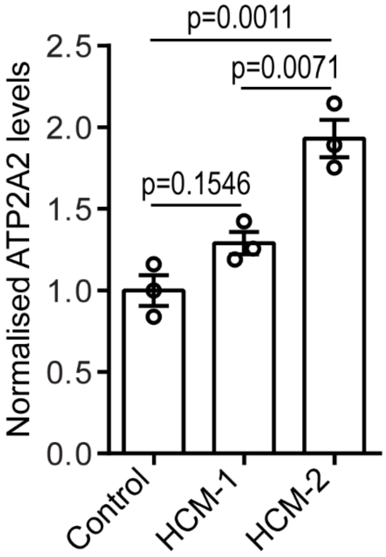

Supplementary figure 5: Graph presented as mean ± s.e.m. showing *ATP2A2* (SERCA) transcript levels in control- and HCM-CMs (n=3 independent experiments; One-way ANOVA followed by Tukey's post-hoc test).

Supplementary figure 6

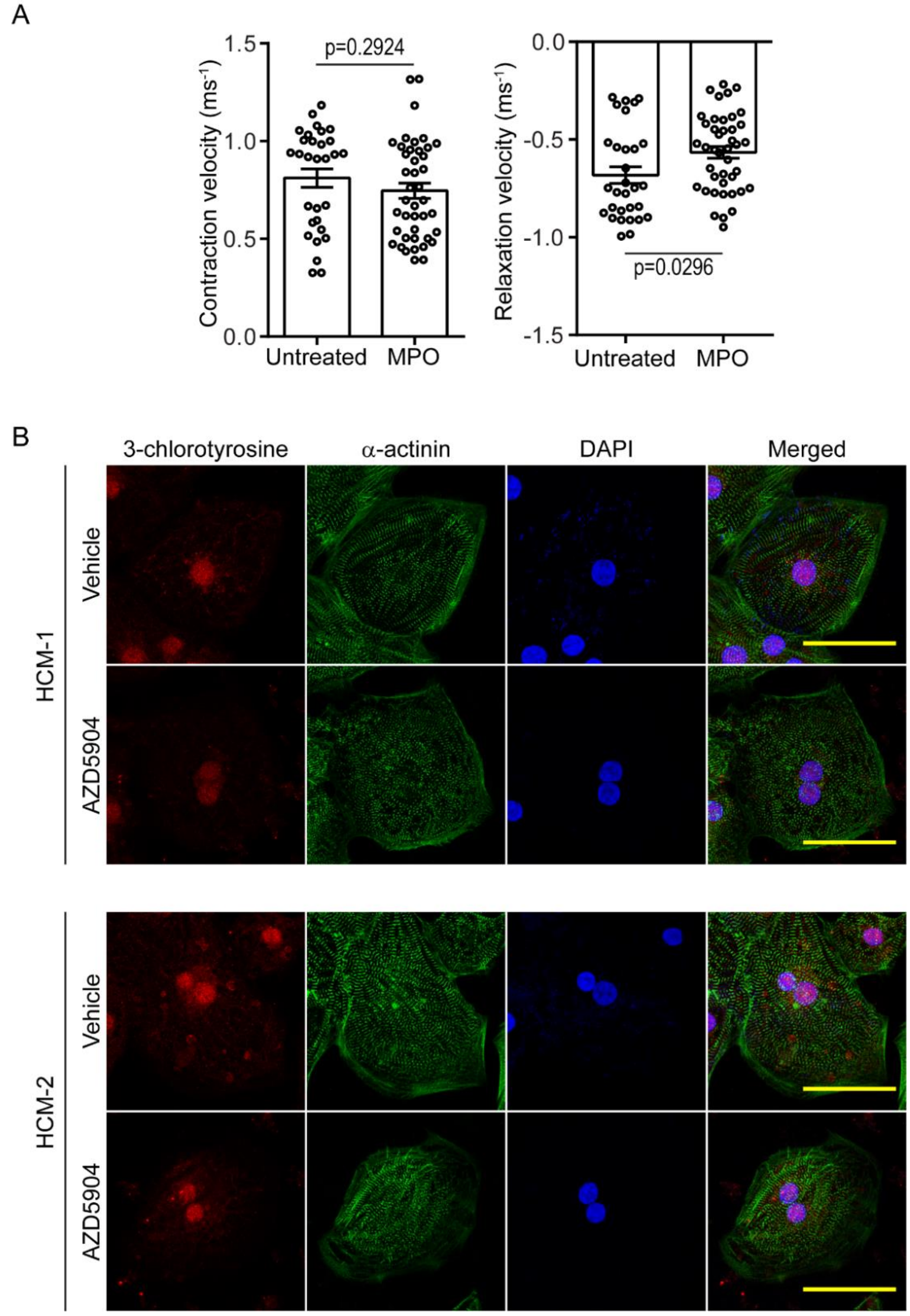

Supplementary figure 6: (A) Graphs presented as mean ± s.e.m. showing comparison in contraction velocity and relaxation velocity in control-CMs pre- and post-MPO treatment (Vehicle n=30; MPO n=42; Unpaired t-test with Welch's correction; 3 independent experiments). (**B**) Single channel and merged pictographs of HCM-1 and HCM-2 pre- and post-AZD5904 treatment stained against 3-chlorotyrosine and sarcomeric α-actinin and counterstained with DAPI. Scale bar: 50µm.

Supplementary figure 7

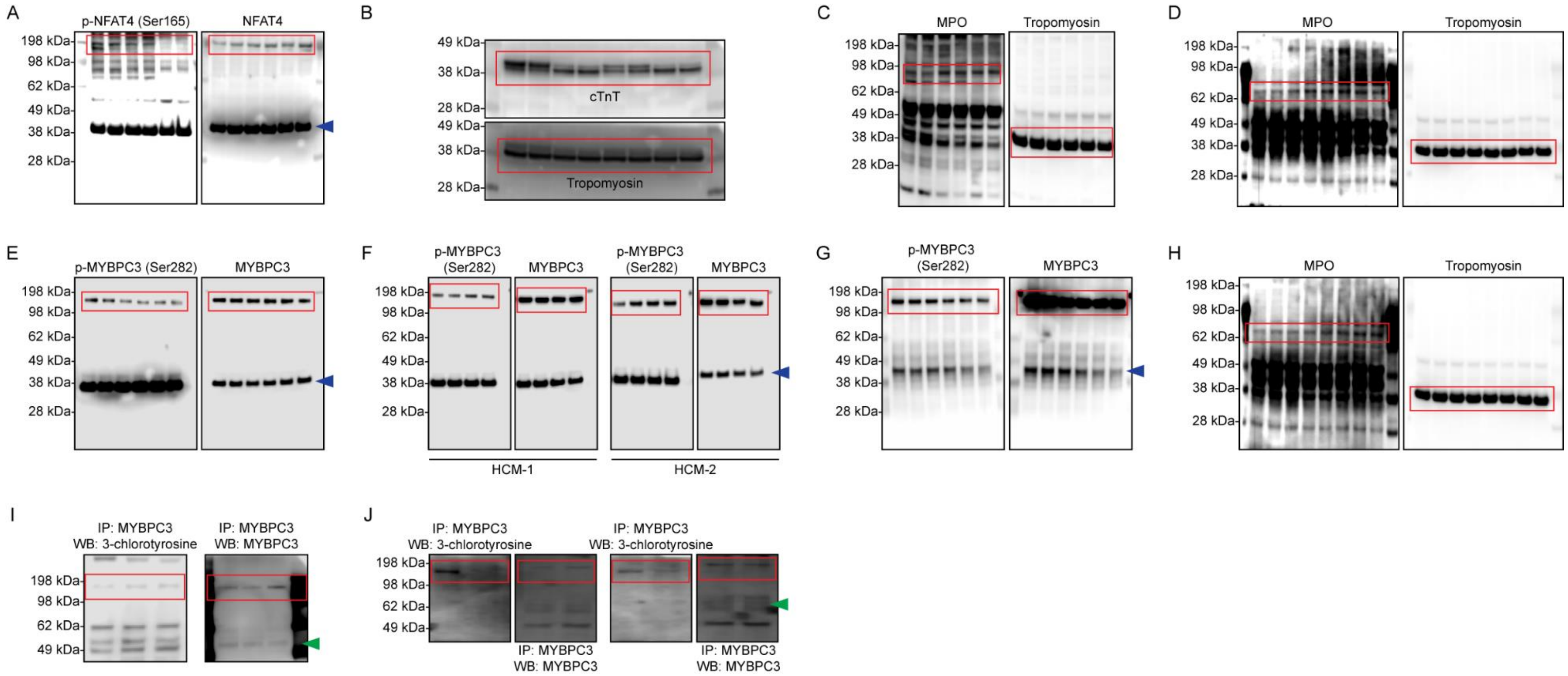

Supplementary figure 7: Complete western blot gels used in the study. Red inset represents cropped region, blue arrowheads indicate respective loading controls and green arrowheads represent IgG heavy chains. The gels correspond with (**A**) Figure 1G, (**B**) Figure 1H, (**C**) Figure 3B, (**D**) Figure 4E, (**E**) Figure 6A, (**F**) Figure 6B, (**G**) Figure 6C (**H**) Figure 6D, (**I**) Figure 6F and (**J**) Figure 6G.

**Supplementary table 1:** Antibodies used in the study		
**Target antigen**	**Host species**	**Manufacturer**
Nanog (IF)	Rabbit	Cell Signaling Technology (99399)
Tra-1-81 (IF)	Mouse	Cell Signaling Technology (83321)
α-actinin (IF)	Mouse	Sigma-Aldrich (A7811)
MLC-2v (IF)	Rabbit	Synaptic Systems (310 003)
NFAT4 (IF)	Mouse	Abcam (ab219063)
NFAT4 (WB)	Rabbit	Abcam (ab245503)
p-NFAT4(Ser-165) (WB)	Rabbit	Abcam (ab59204)
Cardiac troponin T (WB)	Rabbit	Cell Signaling Technology (5593)
MPO (IF, WB, IHC)	Rabbit	Abcam (ab65871)
Tropomyosin (WB)	Rabbit	Cell Signaling Technology (3910)
p-MYBPC3(Ser-282) (WB)	Rabbit	Enzo Life Sciences (ALX-215-057-R050)
MYBPC3 (WB, IP)	Rabbit	Abcam (ab108522)
Chlorotyrosine (IF, WB)	Rabbit	Hycult Biotech (HP5002)
WGA (IF)	Alexa Fluor™ 488 Conjugate	Thermo Fisher Scientific (W11261)
Abbreviations: IF- Immunofluorescence; WB- Western blot; IP- Immunoprecipitation; IHC-Immunohistochemistry		

**Supplementary table 2:** Primers used in the study		
**Target**	**Sense**	**Antisense**
MPO	GGTGATCGGTTTTGGTGGGA	TTAGACACGGTGGTGATGCC
ATP2A2	CGAACCCTTGCCACTCATCT	CCAGTATTGCAGGTTCCAGGT
PXDN	GCAGACCTCCATCCTAGATCTTC	TGCTCCACTAGGTATCCTCTTGA
GAPDH	GTTCGTCATGGGTGTGAACC	GCATGGACTGTGGTCATGAGT
*HCM-1 (*MYBPC3*)	ACACTGGGATTCTGGACTTCAGCTC	GTCCTTCAGCCATTTGACTTGCG
*HCM-2 (*MYH7*)	TGTACACTCCTGAGGTGGTGGCTG	AGTGAAGCCCAGCACATCAAAAGC
*Used for obtaining sequencing information from cDNA		
